# A Dynamic Analysis of Air Pollution: Implications of Economic Growth and Renewable Energy Consumption

**DOI:** 10.3390/ijerph18189906

**Published:** 2021-09-20

**Authors:** Jia Yu Xie, Dong Hee Suh, Sung-Kwan Joo

**Affiliations:** 1 Department of Food and Resource Economics, Korea University, 145 Anam-ro, Seongbuk-gu, Seoul 02841, Korea; ivy-wsns@korea.ac.kr; 2 School of Electrical Engineering, Korea University, 145 Anam-ro, Seongbuk-gu, Seoul 02841, Korea; skjoo@korea.ac.kr

**Keywords:** particulate matter, sulfur dioxide, nitrogen oxide, carbon monoxide, air quality, environmental Kuznets curve, renewable energy, dynamic panel

## Abstract

This paper examines how economic growth and renewable energy consumption are associated with air pollution using a dynamic panel approach. Focusing on several major air pollutants, namely, particulate matter, sulfur dioxide, nitrogen oxide, and carbon monoxide, this paper tests the environmental Kuznets hypothesis and determines whether the use of renewable energy sources contributes to a reduction in air pollution. Data from a balanced panel of 145 countries for the period between 2000 and 2014 was used for the estimation of the dynamic panel model. The results of the dynamic panel model showed inverted U-shaped curves for the relationship between economic development and particulate matter and sulfur dioxide emissions. The results also revealed that increasing renewable energy consumption contributes to an improvement in air quality. Moreover, it was found that urbanization tends to decrease sulfur dioxide and nitrogen oxide emissions, while trade openness reduces particulate matter and carbon monoxide emissions but increases sulfur dioxide emissions.

## 1. Introduction

Air pollution is a global issue because it not only causes global warming but also affects human health. Human activities have exacerbated global warming through the production of excessive greenhouse gases, which induce harmful effects on human health. According to the World Bank and the Institute for Health Metrics and Evaluation [1], about 92% of people worldwide do not breathe clean air due to air pollution, and the resultant costs of air pollution stand at USD 5 trillion every year. Short-term exposure to air pollutants causes dizziness, nausea, asthma, pneumonia, and heart problems [2]. Specifically, exposure to air pollutants affects human skin, causing symptoms such as skin aging, eczema, acne, and urticaria, and affects the eyes, causing irritation or dry eye syndrome [3,4]. Long-term exposure can even induce cancer or death [5]. Particulate matter, benzene, ozone, and dust cause serious damage to the human respiratory system, and some of these substances can even accumulate in cells and blood, having been ingested through the airways [6,7]. Studies have shown that air pollution could be the main cause of cardiovascular disease and affect the immune system extensively [8,9].

As air pollution is known to vary with the rate of industrialization or structural change in economy, and many previous studies have explored how air pollution is related to the stages of economic growth. Grossman and Krueger [10] first found a nonlinear inverted U-shaped curve between per capita income and air pollution, showing that air quality tends to get worse as economies grew until the gross domestic product (GDP) per capita reaches a certain level, after which it gradually improves. They found a tendency for early economic development to neglect ecological protection, and for industrialization processes to cause serious environmental pollution. Many subsequent studies confirmed the inverted U-shaped curve [11,12,13,14,15,16], and subsequently the literature was extended to find an N-shaped curve representing the relationship between economic growth and air pollution [17,18,19,20,21,22]. Most past studies emphasized that sufficient economic growth is required before air pollution problems are addressed.

Moreover, energy consumption has been considered a key factor of driving air pollution. Past studies have focused on the effects of using fossil fuels on air pollution, but the use of renewable energy sources recently became of interest for policy makers because these are environmentally friendly energy sources with potential for sustainable development. Due to the interest in renewable energy, consumption increased dramatically from 520,370.4 ktoe (approximately 7.4% of total energy consumption) in 2000 to 902,546.4 ktoe (approximately 9.7% of total energy consumption) in 2014, according to the International Energy Agency (IEA). In addition, the 2015 Paris Agreement on climate change motivated many countries to develop renewable energy policies. Renewable energy consumption is beneficial for environment: many studies have confirmed that increasing renewable energy intensity improves environmental quality and leads to a decline in carbon dioxide emissions [23,24,25,26,27,28,29]. However, with regard to air pollution, only a few studies in the literature have found evidence that the use of renewable energy sources can ease air pollution problems [23,24,28,30]. 

Due to the importance of economic growth and renewable energy consumption in addressing the problem of air pollution, this paper pursues a deeper understanding of the nexus between air quality, economic growth, and renewable energy consumption. Since the literature has paid little attention to the dynamics of air pollution at a global level, this paper contributes to understanding how air pollution varies dynamically with both economic growth and renewable energy consumption. The specific objective of this paper is as follows. First, it evaluates the dynamic adjustment of air pollution, which shows how fast air pollution changes over time. Second, it examines how economic growth is associated with air pollution. With regard to major air pollutants such as particulate matter, sulfur dioxide, nitrogen oxide, and carbon monoxide, this paper tests the environmental Kuznets hypothesis that economic growth initially increases the levels of these pollutants and then reduces them. Third, this study examines the effects of renewable energy consumption on air pollution. Since the existing literature focuses mainly on analyzing the relationship between carbon dioxide emissions and renewable energy consumption at the single-country or regional levels, this paper contributes to determining whether the use of renewable energy sources can contribute to reducing air pollution at a global level. Fourth, it examines how air pollution is associated with urbanization and trade openness. Since the literature points out that urbanization and trade openness occur in economic development stages, this paper contributes to understanding how they affect air pollution levels.

## 2. Literature Review

An extensive literature review was conducted, and the results are presented in Appendix A. Grossman and Krueger [10] first investigated the impact of the North American Free Trade Agreement (NAFTA) on pollutant emissions. They found that sulfur dioxide and smoke increased with per capita GDP at low national income levels but decreased with economic growth at high national income levels. Later, Panayotou [31], focusing on deforestation, sulfur dioxide, nitrogenous oxides and solid particulate matter as pollution indicators, confirmed the inverted U-shaped curve. Scholars who agreed with the environmental Kuznets curve (EKC) hypothesis believed that in the early stage of economic growth, energy consumption increased due to the scale effect, which led to increased emissions of pollutants. When the economy grew to a certain stage, energy consumption reduced due to the technology effect, resulting in a reduction in the emission of pollutants. At this stage, the use of renewable energy and increasing in consumer demand for clean products can also lead to a reduction in emissions.

The existing literature on air pollution focuses mainly on specific regions and countries. In recent studies, To et al. [32] employed the panel cointegration model to examine emerging markets in Asian regions over the 1980–2016 period, and found that the EKC was generally valid across the regions. As for country-specific studies, Fang et al. [33] investigated the EKC in cubic settings for the linkage between carbon dioxide emissions and economic growth across three regions in China. Tutulmaz [34] investigated linear, quadratic and cubic relationships between carbon dioxide emissions and per capita GDP in Turkey over a period of 40 years, showing that environmental pressure and economic development displayed an inverted U-shaped curve relationship. While many empirical studies found inverted U-shaped curves [14,16,35,36,37,38,39,40,41,42,43], linear and cubic relationships between income and pollution were also supported by a number of studies [14,18,32,33,34,44,45,46,47,48,49].

Regarding energy sources, past studies have focused mainly on fossil fuel consumption, but interest has recently shifted to renewable energy sources, which are seeing increasing use [50]. Many studies have confirmed that increasing renewable energy intensity decreases carbon dioxide emissions [23,24,25,26,27,28,29,51,52]. However, only a few have tested whether the use of renewable energy sources can ease air pollution. For instance, Boudri et al. [24] examined how the use of renewable energy sources would be effective in air pollution abatement in China and India for the period of 1990–2020, and they confirmed that using renewable energy could cut the costs of reducing sulfur dioxide emissions. Zhu et al. [30] tested the effectiveness of renewable energy in improving air quality using a panel dataset covering China’s 31 provinces from 2011 to 2017. Their results indicated that technological innovations in renewable energy would be beneficial for alleviating nitrogen oxide emissions and respirable suspended particle concentrations, but were not significantly associated with a reduction in sulfur dioxide emissions.

Other variables are also considered driving factors of air pollution. The literature mainly outlines the environmental effects of urbanization and trade openness [16,29,35,42,53,54,55,56,57,58,59,60,61,62]. Urbanization refers to the transformation of a country from a rural–agricultural base to an urban–industrial base, which is a key stage in its economic development. [55]. The process of urbanization is typically accompanied by a high degree of resource consumption and serious discharge of pollutants [53]. In addition, urbanization promotes the development of large-scale infrastructure construction and transportation, and in turn, encourages the rapid development of energy-intensive industries [61]. Xie et al. [42] clarified that there is a clear inverted U-shaped relationship between economic growth and fine particulate matter concentrations, and found that population density, industrialization, urbanization and the development of transportation contributed to an increase in fine particulate matter emissions.

Regarding trade openness, the literature indicates that the effects of trade openness on pollution can be divided into three aspects: the scale effect (size of the economy), the composition effect (specialization), and the technology effect (production methods) [35,54]. The scale effect means that an increase in trade volume affects output and energy consumption, and thus carbon dioxide emissions. The composition effect is related to the redistribution of a country’s trade commodity basket, so energy use and environmental quality may increase or decrease depending on whether the country’s specialized industries require more or less energy. The technology effect implies that trade liberalization leads to an improvement in the environment as better technology is applied to produce goods and use energy more efficiently. Cole and Elliott [35] and Antweiler et al. [54] found trade openness is beneficial for environment, and Kacar and Kayalica [16] found that trade openness could reduce sulfur emissions. Salim et al. [29] also found that renewable energy, urbanization, and trade liberalization could reduce pollutant emissions.

In relation to the literature, as detailed in Appendix A, this study differs in three aspects from the previous studies. First, it covers major air pollutants with an extensive panel dataset. The panel dataset reflects the countries that have different air quality at varying economic growth stages. Second, it evaluates the dynamic adjustment of air pollution using the dynamic panel model. Estimating the adjustment speed of air pollution helps us to understand how fast air pollution changes with economic growth, renewable energy consumption, urbanization, and trade openness. Third, this paper focuses the effects of economic growth and renewable energy consumption on air pollution. Since there are few studies that focus on the relationships of air pollution with both economic growth and renewable energy consumption, this study contributes to policies focused on the energy transition from fossil fuels to renewable energy sources at varying economic growth stages.

## 3. Methodology

### 3.1. Dynamic Panel Model 

To test our research hypotheses, we first constructed a panel model for air pollution, which included the following explanatory variables: gross domestic product (GDP) per capita, the ratio of renewable energy consumption to total energy consumption (REC), urbanization (URB) and trade openness (TRA). We also added the squared term of the GDP variable to determine whether there is an inverted U-shaped curve. The conventional fixed-effect panel model is specified as follows.

(1) $$y_{it}=\beta x^{\prime }{}_{it}+v_{t}+u_{i}+\varepsilon_{it},$$

where subscript *i* denotes country and *t* denotes year. The dependent variables include particulate matter 2.5 average exposure (PMA), sulfur dioxide (SO_2_), nitrogen oxide (NO_X_) and carbon monoxide (CO) emissions. In Equation (1), $u_{i}$ represents the individual fixed-effect term, $v_{t}$ indicates the time fixed-effect term, and $\varepsilon_{it}$ denotes the idiosyncratic error term. As the air pollution indicators, GDP and its squared terms are expressed in natural logarithmic form, their estimated coefficients can be interpreted as elasticities.

While we estimated the two-way fixed effect model as a baseline, we also employed the dynamic panel model, which introduces the lagged variable to the original static panel model as follows:

(2) $$y_{it}=\delta y_{i,t-1}+\beta x^{\prime }{}_{it}+\mu_{it}$$

where δ is the autoregressive parameter to be estimated. In Equation (2), $\mu_{it}$ is the composite error term, $\mu_{it}=u_{i}+\varepsilon_{it}$ where $u_{i}$ is the unobservable individual effect and $\varepsilon_{it}$ is the random disturbance.

Since traditional regression techniques produce biased and unreliable estimates when the interference term is correlated with endogenous variables, Anderson and Hsiao [63] introduced the first differencing of the model to Equation (2). The differential dynamic panel model is constructed as follows:

(3) $$∆y_{it}=\delta ∆y_{i,t-1}+\beta ∆x^{\prime }{}_{it}+∆\varepsilon_{it}$$

where $∆y_{i,t-2}$ is used as an instrument for $∆y_{i,t-1}$. As instrumental variable estimation leads to consistent but not necessarily efficient estimates [64], the generalized method of moments (GMM) developed by Arellano and Bond [65] is employed to include additional instruments and all the available moment conditions. The GMM performs a first-order difference to remove the influence of the fixed effects, and then uses a set of lagged explanatory variables as instrumental variables for the corresponding variables in the difference equation.

### 3.2. Data Description

A panel dataset covering 145 countries over the period from 2000 to 2014 was used for our empirical analyses. Air pollution was represented by particulate matter 2.5 average exposure (PMA), sulfur dioxide (SO_2_), nitrogen oxide (NO_X_) and carbon monoxide (CO) emissions. The yearly data for PMA were obtained from the Environmental Performance Index (EPI) compiled by Yale University (2016), and those for SO_2_, NO_X_, and CO were obtained from the Community Emissions Data System (CEDS). PMA emissions were measured in $\mu g/m^{3}$, whereas the emissions of SO_2_, NO_X_, and CO were measured in $t/km^{2}$.

The explanatory variables were renewable energy consumption (REC), gross domestic product (GDP) per capita, urbanization (URB), and trade openness (TRA). The yearly data for renewable energy consumption rate (the ratio of renewable energy consumption to total final energy consumption), GDP per capita (constant 2010 USD), urbanization rate (the ratio of urban population to total population), and trade openness (the ratio of total trade to GDP) were obtained from the World Development Indicator of the World Bank. Detailed descriptions of the variables are listed in Table 1.

## 4. Results

The estimation results for the major air pollutants are shown in Table 2, Table 3, Table 4 and Table 5. While the results of the fixed-effect model are reported for the static results, our interpretations are based on the results of the dynamic panel model. Overall, the estimates of the lagged variables of the air pollutants represent the dynamics of air pollution. The estimates for PMA, SO_2_, NO_X_, and CO were 0.43, 0.88, 0.93, and 0.83, respectively, implying that the adjustment of most air pollutants is sluggish; the speed of change for PMA is faster than in the other air pollutants.

Regarding the relationship between economic growth and air pollution (Table 2, Table 3, Table 4, and Table 5), the results show that inverted U-shaped curve relationships exist between economic growth and the air pollutants PMA and SO_2_. While the results of the fixed-effect model confirm the EKC for all the examined air pollutants, those of the dynamic panel model only confirm the existence of the EKC for PMA and SO_2_. The different results obtained using the dynamic panel model can be attributed to the addition of the dynamic term and addressing the endogeneity problem, thus providing more rigorous estimates. In Table 2 and Table 3, the results for PMA and SO_2_ imply that more air pollutants are emitted as the economy grows until the GDP per capita reaches a certain level, after which emissions gradually improve. However, the results for NO_X_ and CO emissions show that these continue to increase with economic growth, a pattern which can be attributed to a continuous increase in the ownership automobiles, since vehicle engines are a major source of nitrogen oxide and carbon monoxide.

Using the peak of the inverted U-shaped curve, we determined the level of per capita GDP that correlated with a reduction in pollutant emissions. The per capita GDP level for peak PMA emissions was approximately USD 12,752.47, and that for SO_2_ was approximately USD 11,249.74. The sources of particulate matter 2.5 are more diverse because they are formed in various combustion processes. However, sulfur dioxide is generated mostly by the burning of coal or oil in power plants and by factories that produce chemicals, paper, and steel. Most economies generally have a large number of factories reliant on burning coal and oil in their early stage of development, but tend to move away from resource-dependent manufacturing when they achieve sufficient economic growth. 

Moreover, our results show that renewable energy consumption has negative effects on air pollution, which indicates that increasing renewable energy consumption can improve air quality. The use of renewable energy reduces SO_2_ and NO_X_ emissions, but it is not associated closely with PMA and CO emissions. The use of renewable energy does not contribute to reducing PMA, a finding which can be attributed to the diversity of sources generating PMA emissions. CO emissions are related to the incomplete combustion of fuels. Although the use of renewable energy directly reduces the use of fossil fuels, their inefficient use still generates CO emissions. However, despite the limited evidence of any effect on PMA and CO emissions, the results show the potential contribution of renewable energy sources to improving air quality via the reduction of SO_2_ and NO_X_ emissions.

The results indicate that urbanization tends to decrease SO_2_ and NO_X_ emissions. This can be attributed to the fact that urbanization induces economies to move away from the industrial and manufacturing sectors. Urbanization also transforms rural energy structures dominated by solid fuels into urban energy structures dominated by clean fuels, a process which contributes to the reduction of SO_2_ and NO_X_ emissions. Regarding trade openness, the results show that PMA and CO emissions improve as openness increases, but SO_2_ emissions deteriorate. As discussed in [16,27,29], more frequent trade activities increase the intensity of production, and lead to a greater consumption of fossil fuels for transportation and trade, which increases SO_2_ emissions. On the other hand, developed countries tend to gradually transfer their high-emission manufacturing activities to underdeveloped and developing countries. In addition, trade accelerates the exchange of technologies that can improve energy efficiency, thereby reducing the incomplete combustion of fossil fuels, which leads to reductions in PMA and CO emissions.

## 5. Discussion

**Figure 1:**
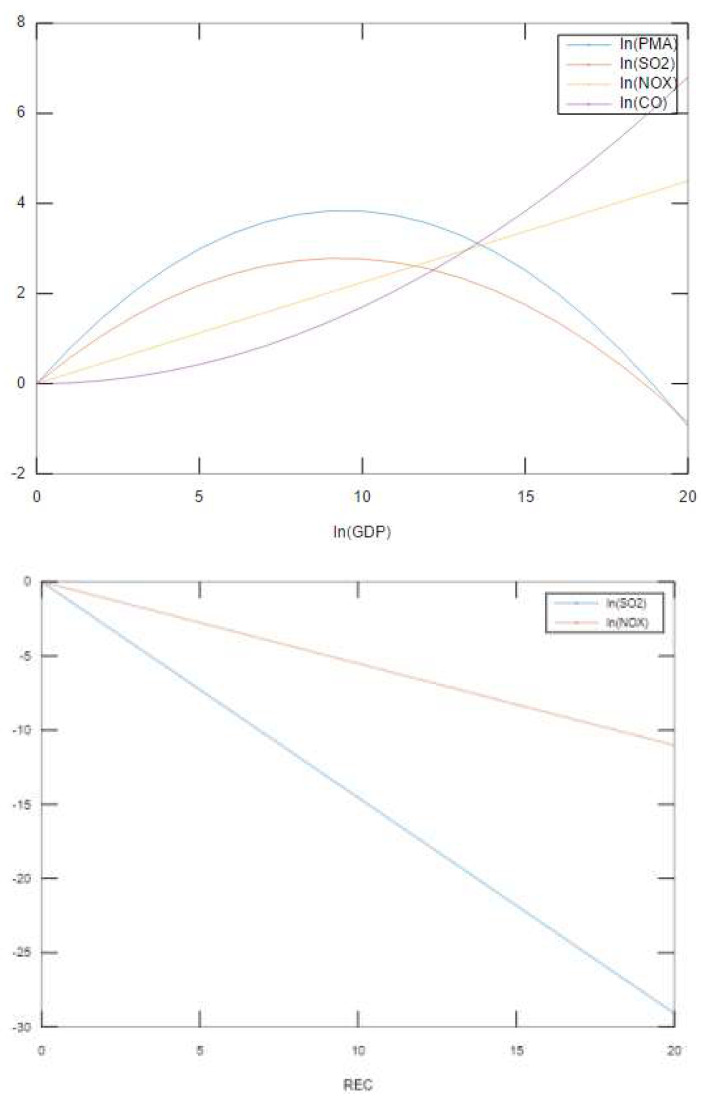

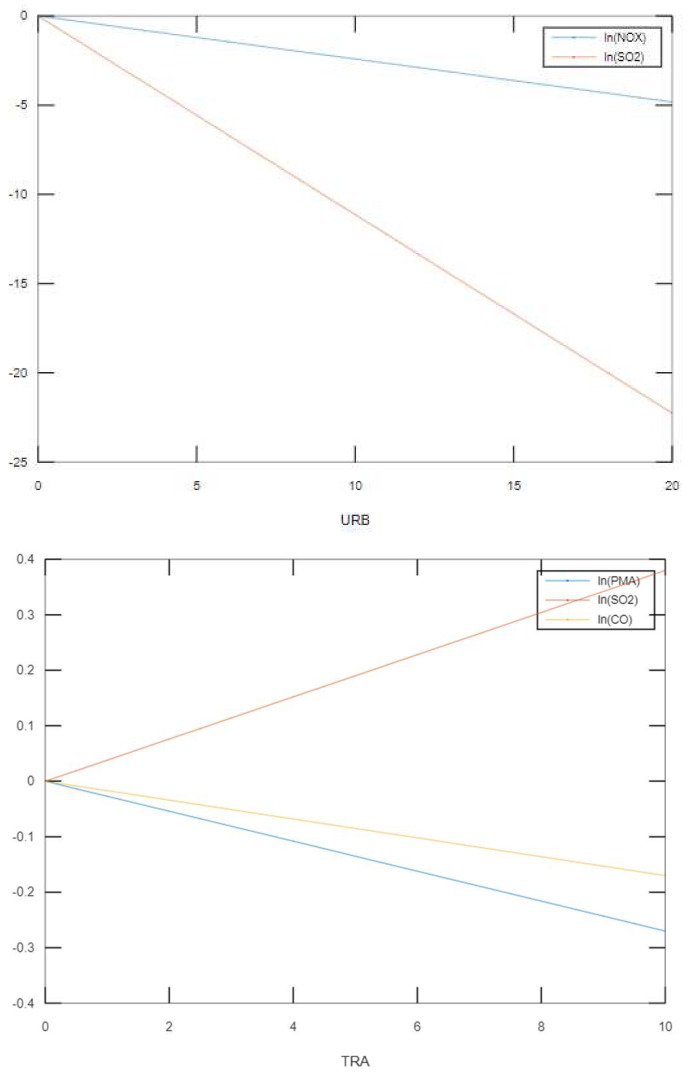
Graphical Summary of Results.

The major findings are summarized in Figure 1. While NO_X_ and CO emissions continue to increase linearly and nonlinearly with economic growth, PMA and SO_2_ emissions first increase mainly due to the industrialization, and then decline after economy achieves a certain level of income. The findings show that underdeveloped or developing countries can suffer from air pollution, but they can, at least, alleviate air pollution problems related to PMA and SO_2_ emissions if their per capita GDP reaches about USD 11,000 to 12,000. Since the early stage of economic growth tends to require more combustion of coal, low-income countries inevitably release high levels of air pollutants. As income grows, countries tend to improve energy efficiency and shift to nuclear generation, which generates less pollution in the form of PMA and SO_2_ emissions. Moreover, consumers’ demands for eco-friendly energy sources and products contribute to reductions in PMA and SO_2_ emissions. However, the results for NO_X_ and CO emissions show that these pollutants continue to increase as economic growth continues, which can be attributed to a continuous increase in the use of automobiles, since combustion engines are the main source of nitrogen oxide and carbon monoxide. Technological improvements such as catalytic reduction and the more complete combustion of fuels are required to reduce NO_X_ and CO emissions as average incomes grow.

More importantly, the findings show that an increase in renewable energy consumption can be a way of alleviating air pollution problems effectively. The energy transition from fossil fuels to renewable energy sources can contribute to reductions in SO_2_ and NO_X_ emissions. The findings on the impacts of economic growth and of renewable energy consumption on pollution suggest that we should not simply wait for economic growth to address air pollution problems. It can be seen that although low-income countries tend to suffer from air pollution, the implementation of active policy measures to increase the use of renewable energy can improve air quality, regardless of economic growth. Moreover, the use of renewable energy can help high-income countries reduce SO_2_ and NO_X_ emissions. While the use of renewable energy can accelerate the speed at which SO_2_ emissions reduce with economic growth, it can reduce NO_X_ emissions directly.

Some policy measures can be formulated to promote the consumption of renewable energy. Since high-income countries have more advanced renewable energy technology than low-income countries, they can diffuse these innovations, and the resultant improvements in air quality, to low-income countries. Moreover, strict regulations on energy sustainability can be imposed, and public awareness of the importance of renewable energy can be raised [25]. Governments can strengthen the measures used to control pollution and strictly enforce environmental legislation affecting industries which are highly polluting and consume high levels of energy. In addition, they can encourage and subsidize technological innovations that reduce the cost of renewable energy [52]. For consumers, they can also enhance public awareness of environmental protection, encourage residents to use public transportation instead of private cars to travel, and encourage residents to use energy-saving or renewable energy appliances [51].

Our findings also show that urbanization tends to decrease SO_2_ and NO_X_ emissions. In line with the findings of Lin and Zhu [62], urbanization reduces air pollutant concentrations due to the transition to advanced urban infrastructure and public transportation systems. The construction of urban rail transit can greatly reduce dependence on private cars and increase people’s awareness of environmental protection. In addition, urban greening, low-carbon infrastructure, and green transportation systems can reduce the concentration of air pollutants [28]. For instance, advanced technology can be applied in civil architecture to improve energy efficiency and reduce emissions [66], and the use of solar lighting and new-energy vehicles can mitigate the dependence of the auto industry on petroleum [67]. Combined with the impact of renewable energy on SO_2_ and NO_X_ emission indicated in our results, urbanization can contribute further to reductions in emissions.

Regarding the findings for trade openness, as discussed by Managi et al. [56], previous studies have been largely inconclusive with regard to the overall impact of trade on the environment. In theory, trade openness influences emissions through the composition effect, which includes environmental regulation and the capital–labor effects. If a country has less stringent environmental regulations and a high capital-to-labor ratio, it may produce more emissions. The relative size of the two effects will determine whether the composition effect is positive or negative [34,54,56]. Since our findings indicate that trade openness raises SO_2_ emissions, more stringent environmental regulation should be formulated to force capital-intensive industries to reduce their emissions as income grows.

## 6. Conclusions

The main purpose of this paper is to study the nexus between air quality, economic growth and renewable energy consumption using dynamic analysis. Although there are many studies on the environmental Kuznets curve in the literature, few papers have examined the effects of both economic growth and renewable energy consumption on air pollution. In particular, many studies have demonstrated that the use of renewable energy mitigates carbon dioxide emissions, but few have studied the impact of renewable energy on air pollution. Accordingly, this paper contributes to a deeper understanding the dynamics of air pollution by examining the relationships between air pollution, economic growth, and renewable energy consumption.

A balanced panel of 145 countries, covering the period between 2000 and 2014, was utilized to estimate the dynamic panel model. The results of the fixed-effect model estimates indicated the existence of an EKC relationship between various air pollutants and income, but the estimation results of the dynamic panel model only confirmed the existence of an EKC relationship between PMA and SO_2_ emissions and income. Our results also show that renewable energy consumption and air pollution have a statistically significantly inverse relationship, which indicates that increased renewable energy consumption contributes effectively to improving air quality. It was also found that urbanization tends to decrease SO_2_ and NO_X_ emissions, while trade openness reduces PMA and CO emissions but is associated with higher SO_2_ emissions.

The main findings indicate that air pollution is affected by both economic growth and renewable energy consumption. It is important to stimulate economic growth to address air pollution problems, since countries in the early stage of economic growth inevitably face air pollution problems due to economic activities that rely on fossil fuels. The findings are consistent with the EKC hypothesis, suggesting that economic growth should not be viewed as a threat to environmental quality. As raising per capita income is the most reliable way to solve environmental problems, it is possible to solve the negative external effects brought about by future economic growth when income increases [68]. Webber and Allen [69] also believe that economic growth will eventually lead to the improvement of the environment, while environmental protection policies will only slow down economic growth.

The findings also support the suggestion that degradation of air quality can be reversed if the economy encourages producers to adopt new technologies that reduce air pollution and if consumers demand more environmentally friendly products. Moreover, the findings suggest that countries can alleviate air pollution by increasing the use of renewable energy. Although countries in the early stages of economic growth tend to use fossil fuels more, the resultant degradation of air quality can be reduced via a significant transition from fossil fuels to renewable energy. As such, governmental policies that aim for economic growth alongside the use of renewable energy may be beneficial for addressing economic, social, and environmental problems related to air quality.

## Figures and Tables

**Table 1:** Descriptive Statistics of Variables.

Variable	Mean	Std. Dev.	Min.	Max.
PMA $\left(\mu g/m^{3}\right)\;$	8.78	6.31	0.10	50.30
SO_2_ $\left(t/km^{2}\right)\;$	11.59	129.72	0.00	2069.88
NO_X_ $\left(t/km^{2}\right)$	1.89	3.35	0.01	27.13
CO $\left(t/km^{2}\right)$	9.07	14.87	0.12	129.33
GDP ($)	11,649.26	17,304.65	221.10	111,968.30
REC (ratio)	0.34	0.30	0.00	0.97
URB (ratio)	0.55	0.22	0.08	0.97
TRA (ratio)	0.82	0.42	0.00	3.93

Note: PMA is particulate matter 2.5 average exposure; SO_2_ is sulfur dioxide; NO_X_ is nitrogen oxide; CO is carbon monoxide; GDP is per-capita gross domestic product; REC is renewable energy consumption; URB is urbanization; TRA is trade openness. Sources: Environmental Performance Index (EPI); Community Emissions Data System (CEDS); and World Bank.

**Table 2:** Estimation Results for Particulate Matter 2.5 Average Exposure (PMA).

	Fixed Effect Model	Dynamic Panel Model
$\ln PMA_{\;t-1}$	-	0.433 ***
		(0.007)
lnGDP	1.546 ***	0.813 ***
	(0.345)	(0.109)
${\left(\ln GDP\right)}^{2}$	−0.086 ***	−0.043 ***
	(0.021)	(0.006)
REC	−0.151 **	−0.036
	(0.193)	(0.033)
URB	0.811 ***	0.190
	(0.540)	(0.125)
TRA	−0.034	−0.027 ***
	(0.049)	(0.007)
Constant	−5.196 ***	-
	(1.431)	

Notes: *** and ** denote the level of significance at 1%, 5% and 10% respectively; numbers in parentheses are robust standard errors.

**Table 3:** Estimation Results for Sulfur Dioxide Emissions (SO_2_).

	Fixed Effect Model	Dynamic Panel Model
$\ln SO_{2}{}_{\;t-1}$	-	0.883 ***
		(0.014)
lnGDP	3.017 ***	0.597 **
	(0.561)	(0.258)
${\left(\ln GDP\right)}^{2}$	−0.185 ***	−0.032 **
	(0.039)	(0.015)
REC	−3.175 ***	−1.454 ***
	(0.527)	(0.086)
URB	1.628 ***	−1.113 ***
	(0.988)	(0.218)
TRA	−0.185 **	0.038 ***
	(0.099)	(0.016)
Constant	−12.392 ***	-
	(2.286)	

Notes: *** and ** denote the level of significance at 1%, 5% and 10% respectively; numbers in parentheses are robust standard errors.

**Table 4:** Estimation Results for Nitrogen Oxide Emissions (NO_X_).

	Fixed Effect Model	Dynamic Panel Model
$\ln NOX_{\;t-1}$	-	0.931 ***
		(0.010)
lnGDP	1.275 ***	0.225 ***
	(0.438)	(0.108)
${\left(\ln GDP\right)}^{2}$	−0.071 ***	−0.006
	(0.028)	(0.006)
REC	−1.701 ***	−0.551 ***
	(0.308)	(0.049)
URB	1.482 ***	−0.241 ***
	(0.751)	(0.088)
TRA	−0.126 ***	−0.008
	(0.053)	(0.006)
Constant	−6.129 ***	-
	(1.788)	

Notes: *** denotes the level of significance at 1%, 5% and 10% respectively; numbers in parentheses are robust standard errors.

**Table 5:** Estimation Results for Carbon Monoxide Emissions (CO).

	Fixed Effect Model	Dynamic Panel Model
$\ln CO_{\;t-1}$	-	0.829 ***
		(0.008)
lnGDP	1.645 ***	−0.132
	(0.431)	(0.102)
${\left(\ln GDP\right)}^{2}$	−0.095 ***	0.017 ***
	(0.095)	(0.006)
REC	−0.785 ***	−0.025
	(0.328)	(0.044)
URB	1.126 ***	0.062
	(0.717)	(0.091)
TRA	−0.086 ***	−0.017 ***
	(0.059)	(0.006)
Constant	−5.699 ***	-
	(1.783)	

Notes: *** denotes the level of significance at 1%, 5% and 10% respectively; numbers in parentheses are robust standard errors.

## Data Availability

The data are available publicly from the Environmental Performance Index of Yale University, the Community Emissions Data System, and the World Development Indicator of the World Bank.

## Appendix A

**Table A1:** Literature Review.

Authors	Pollution	Regions and Periods	Findings	Methodology
Grossman and Krueger (1991) [10]	SO_2_ and smoke	42 countries (1977–1988)	Inverted U-shaped curve of SO_2_ and smoke	Random effect model
Shafik (1994) [12]	SO_2_, SPM, CO_2_	149 countries (1960–1990)	Inverted U-shaped curve of SO_2_, SPM; monotonically increasing CO_2_	OLS
Welsch (2004) [14]	SO_2_, NO_2_, SPM	106 countries (1980–2000)	Inverted U-shaped curve of CO_2_	OLS, SUR
Kacar (2014) [16]	SO_2_	20 countries (1951–2000)	Inverted U-shaped curve of SO_2_	OLS, Fixed effect model, Random effect model
Torras (1998) [18]	SO_2_, smoke and heavy particles	58 countries (1977–1991)	N-shaped curve of SO_2_ and smoke; inverted N-shaped curve of heavy particles	OLS
Sulaiman (2013) [25]	CO_2_	Malaysia (1980–2009)	Inverted U-shaped curve; renewable energy consumption reduces CO_2_ emissions	ARDL model; VEC model
Bilgili et al. (2016) [26]	CO_2_	17 OECD countries (1977–2010)	Inverted U-shaped curve; renewable energy consumption reduces CO_2_ emission	FMOLS; DOLS
Jebli et al. (2016) [27]	CO_2_	25 OECD countries (1980–2010)	Inverted U-shaped curve; renewable energy consumption reduces CO_2_ emission	FMOLS; DOLS
Salim et al. (2017) [29]	CO_2_	86 Asian developing countries (1980–2010)	Inverted U-shaped curve; renewable energy consumption reduces CO_2_ emission	MG; CCEMG
Zhu et al. (2020) [30]	SO_2_, NO_X_, PM10	31 provinces in China (2011–2017)	Inverted U-shaped curve of SO_2_; renewable energy consumption reduces NOX, PM10 emissions	Spatial econometric model
Panayotou (1993) [31]	Deforestation, SO_2_, NO_X_ and SPM	68 countries for deforestation; 55 countries for SO_2_, NO_X_ and SPM (1980)	Inverted U-shaped curve of deforestation, SO_2_ and NOX	OLS
To et al. (2019) [32]	CO_2_	25 emerging markets and developing countries in Asia (1980–2016)	Inverted N-shaped curve	FMOLS; DOLS
Fang et al. (2019) [33]	CO_2_	30 provinces of China (1995–2016)	Inverted N-shaped curve	Fixed effect model, Random effect model
Tutulmaz (2015) [34]	CO_2_	Turkey (1968–2007)	Monotonically increasing; inverted U-shaped curve	Co-integration
Cole et al. (1997) [35]	SO_2_, NO_X_, CO and SPM	OECD countries (1960–1991)	Inverted U-shaped curve of SO_2_, NO_X_, CO and SPM	GLS
Xu and Lin (2015) [37]	CO_2_	China (2000–2012)	Inverted U-shaped curve of CO_2_	Dynamic nonparametric additive regression model
Sephton and Mann (2016) [38]	SO_2_ and CO_2_	UK (1830–2003)	Inverted U-shaped curve of SO_2_ and CO_2_	OLS
Och (2017) [39]	NO_X_	Mongolia (1981–2012)	U-shaped curve	Co-integration and VECM model
Sapkota and Bastola (2017) [40]	CO_2_	14 Latin American countries (1980–2010)	Inverted U-shaped curve; U-shaped curve	Fixed effect model, Random effect model
Wang et al. (2017) [41]	CO_2_, N_2_O and methane	U.S. (1960–2010)	U-shaped curve of methane	Co-integration and correlation methods
Xie et al. (2019) [42]	PM2.5	249 Chinese cities (2015)	Inverted U-shaped curve	Semiparametric spatial autoregressive model
Haider et al. (2020) [43]	N_2_O	31 countries (1981–2012)	Inverted U-shaped curve	Co-integration and correlation methods
Richmond and Kaufmann (2006) [44]	CO_2_	36 countries (1973–1997)	Monotonically increasing	OLS
Abdallah et al. (2013) [45]	CO_2_	Tunisia (1980–2010)	N-shaped curve	VECM model
Onafowora and Owoye (2014) [46]	CO_2_	8 countries (1971–2010)	Inverted U-shaped; Inverted N-shaped	ARDL model
Luo et al. (2014) [47]	PM10, SO_2_, NO_2_	31 provincial capitals of mainland China	Monotonically increasing SO_2_ and PM10; Inverted U-shaped curve of NO_2_	OLS
Akpan (2015) [48]	CO_2_	47 countries (1970–2008)	Monotonically increasing; inverted U-shaped curve; N-shaped curve	OLS; GLS; 2SLS
Baek (2015) [49]	CO_2_	7 Arctic countries (1960–2010)	Inverted U-shaped curve; N-shaped curve	ARDL model

Note: OLS is ordinary least square; SUR is seemingly unrelated regression; ARDL is autoregressive distributed lag; VEC is vector error correction; FMOLS is fully modified OLS; DOLS is dynamic OLS; MG is mean group; CCEMG is common correlated effects mean group; GLS is generalized least square; 2SLS is two-stage least square.
